# Supplementary figures and images for: The miRNA biogenesis in marine bivalves
Source: PeerJ. 2016 Mar 7;4:e1763. doi: 10.7717/peerj.1763 (PMC4793324; doi:10.7717/peerj.1763)

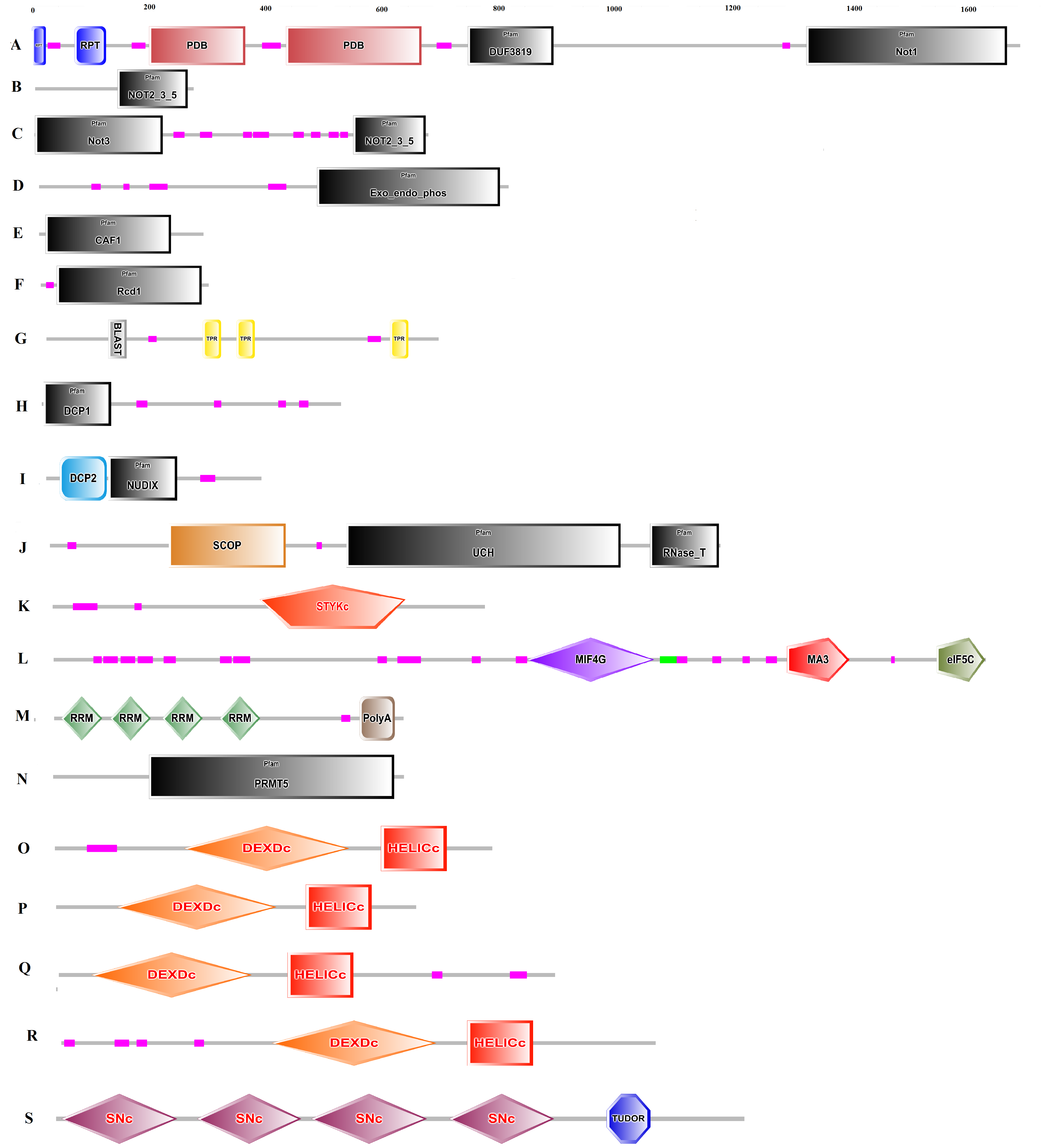

Supplement: File S3 — A, CNOT1; B, CNOT2; C, CNOT3; D, CNOT6; E, CNOT7; F, CNOT9; G, CNOT10; H, DCP1, I, DCP2; J, PAN2; K, PAN3; L, eIF4G; M, PABP; N, PRMT5; O, DDX5; P, DDX6; Q, DDX20; R, DDX42; S, TUDOR-11 and T, MAEL. Domain organization of each protein is shown, green bars represent coiled-coil regions and purple bars disordered regions. Length is expressed as amino acid scale above the figure. [file peerj-04-1763-s003.tif]
